# Reintroduction of the European Capercaillie from the Capercaillie Breeding Centre in Wisła Forest District: Genetic Assessments of Captive and Reintroduced Populations

**DOI:** 10.1371/journal.pone.0145433

**Published:** 2015-12-18

**Authors:** Tomasz Strzała, Artur Kowalczyk, Ewa Łukaszewicz

**Affiliations:** 1 Department of Genetics, Faculty of Biology, Wroclaw University of Environmental and Life Sciences, Wrocław, Poland; 2 Division of Poultry Breeding, Institute of Animal Breeding, Faculty of Biology, Wroclaw University of Environmental and Life Sciences, Wrocław, Poland; Sichuan University, CHINA

## Abstract

The Western capercaillie (*Tetrao urogallus*) is a specific bird species, which, despite its very broad distribution and large global population size, is highly endangered in many Western and Central European countries. According to the species situation, in many countries (including Poland), breeding and reintroduction programmes have been started. One of the most complex and large-scale reintroduction programmes was started in Bory Dolnośląskie Forest, and the Capercaillie Breeding Centre in Wisła Forest District was used as one of the sources of individuals for reintroduction. As genetic tools provide essential knowledge about species biodiversity, which is crucially important during the breeding process and reintroduction, both captive and reintroduced grouse populations were genetically analysed. We were particularly interested in genetic diversity of the individuals in both populations and the genetic relationship between them, as well as between them and other capercaillie representatives from their current range. To fulfil these goals we determined nine microsatellite loci along with a fragment of the mitochondrial control region. Genetic diversity parameters were moderate to high compared to populations from other Central and Western European countries. Both populations were clustered into three distinct genetic clades based on microsatellites. Phylogenetic analysis placed all mitochondrial haplotypes we revealed in the Eurasian clade. The present results will play an important role as they will help to preserve and maximize genetic diversity in captive populations, and will provide a basis for future monitoring of the reintroduction process.

## Introduction

The Western capercaillie (*Tetrao urogallus*) is distributed among Western Palearctic boreal and mountain forests, but in spite of its wide distribution, in many central European countries it is critically endangered [1]. The size of the capercaillie population has been systematically decreasing for over the last 100 years and has resulted in population fragmentation and the loss of many small, isolated populations [2]. The main reasons for the *T*. *urogallus* population decline are degradation of habitats due to improper forest management, disturbance of capercaillie habitats and ecosystems, excessive hunting, industrial pollution and increased density of predators. In Poland, like many other central European countries, the Western capercaillie population decline has been severe. From over 2500 individuals at the beginning of the 20th century, currently the Polish *T*. *urogallus* population is estimated at 380–500 birds, which are located in four isolated populations: Augustowska Primeval Forest, Western Carpathian, Solska Primeval Forest and Bory Dolnośląskie Forest (BDF) [3]. Consequently, the capercaillie in Poland is under multistage protection of its habitats (Natura 2000 project) and the species itself. In 1995 it was entered into the Polish Red Data Book of Animals [4] as a critically endangered species and listed in Appendix I to the 79/409/EEC Bird Directive.

Information about the genetic diversity within species, and in particular within critically endangered species, is important knowledge, crucial for proper management and population restoration [5]. Western capercaillie genetic variability and population genetic structure have been analysed in many European populations [6–14]. In Poland, Rutkowski et al. [9] analysed genetic diversity of all four separate populations and revealed a substantial level of microsatellite polymorphism. The average number of alleles per locus was 4.45, expected heterozygosity was 0.585, and two populations revealed significant heterozygote deficiency, probably due to high population decline (F_IS_ = 0.45 in BDF and Western Carpathian (F_IS_ = 0.36)). Rutkowski et al. [9] concluded that due to population fragmentation and substantial population decline, the proper way of Polish population protection and restoration should consist of implementing the captive breeding programmes combined with habitats improvement and reconstruction. To fulfil those goals, *T*. *urogallus* restitution programmes were undertaken in several Polish forest districts, and in 2009 one was started in Ruszów in the BDF [15].

The number of capercaillie in the BDF decreased from ~360 individuals in the mid-20th century [16] to 18 birds in 2006 [17], and only two incidental observations of females in 2009 [15] were noted. Such a population decline made it impossible to use local individuals in the restoration process. Thus, between 2009 and 2014, 131 capercaillie individuals (75 males and 56 females) were released in Ruszów Forest District (Merta, pers. comm.)– 39 birds from the Breeding Centre of Forest Grouse in Kadzidłowo, 75 individuals from the Capercaillie Breeding Centre in Wisła Forest District (CBC-WFD) and 17 birds from the Capercaillie Breeding Centre in Leżajsk Forest District. Before initiation of the reintroduction process, complex analyses of causes of the capercaillie population decline were performed. In consequence, composite actions for active protection were planned and implemented: (i) use of captive breeding individuals for reintroduction, (ii) reduction and monitoring of predator numbers, (iii) habitat improvement, (iv) anthropopressure reduction and (v) ecological education [18]. Captive reared birds were reintroduced into two historical capercaillie refuges (with a combined area of c. 18 km^2^), where detailed analyses of habitat quality were performed using the Habitat Suitability Index [19], and then habitats were improved to be assessed as ‘good’ [15].

The majority of individuals reintroduced in Bory Dolnośląskie Forest were reared in the CBC-WFD, which was founded in 2002 and is one of the biggest capercaillie breeding stations in Poland [20]. The CBC-WFD started its activity with birds hatched from eggs imported from Belarus (3 males and 7 females). During the next years the captive population was enlarged with eggs collected from nests found in local forest districts as well as by birds which were injured or aggressive and were sheltered by foresters. In 2012 the basic breeding flock consisted of 16 males and 32 females, from which eight (two males and six females) were of Belarusian origin [20]. Both genetic lines (Belarusian and Western Carpathian) were reproduced separately. To improve the breeding success in the CBC-WFD, assisted reproduction techniques were implemented, allowing the number of offspring to be increased [21–24].

The reintroduction process is complex and its success depends on many factors, including the understanding of species ecology, habitat preparation, the number of reintroduced individuals and the reintroduced population’s adaptive potential [5]. Adaptation to the new environment is crucial for the reintroduced population and directly depends on its genetic diversity. Choosing appropriate individuals from a captive population allows one to create a non-distorted, sustainable population that will not excessively deplete the source population. Thus, first the captive and then the reintroduced population should be monitored both with genetic markers and/or with pedigree information (captive population). Such a multidirectional reintroduction process has much more chance of finishing with a wild, self-sustainable population [5].

To meet the goals of the best practices in reintroduction, we analysed the genetic diversity in both the captive (which was one of the source populations for reintroduction) and the new wild populations. We were interested particularly in: (i) the level of microsatellite and mtDNA diversity in captive and reintroduced populations, (ii) the genetic relationship between capercaillie from the CBC-WFD and the BDF revealed by its individuals genotype clustering, and (iii) the phylogenetic relationships between captive and reintroduced individuals with *T*. *urogallus* from other European countries.

## Materials and Methods

### Ethics statement

The National Forestry in Wisła District obtained permission (DOP-OZGIZ.6401.03.171.2011.km, dated on: May 10, 2011; expiry date: December 31, 2021) issued by the General Director of Environmental Protection, signed by Dr Michał Kiełsznia, for keeping, reproduction and collection of the biological materials for experimental purposes, every year up to 50 adult capercaillie (*Tetrao urogallus*) and 150 juvenile birds in the CBC-WFD, Poland. The used protocol was approved by II Local Ethics Commission for Experiments Carried on Animals (Permit: NR 31/2010; issued on February 22, 2010) and authors possess individual permission for carrying the experiments on animals, including capercaillie; Ewa Łukaszewicz—permit: No. 23/2009, Artur Kowalczyk—permit: No. 24/2009, both documents dated on June 19, 2009. During all procedures all efforts were made to minimize animals suffering.

### Sampling

**Fig 1:**
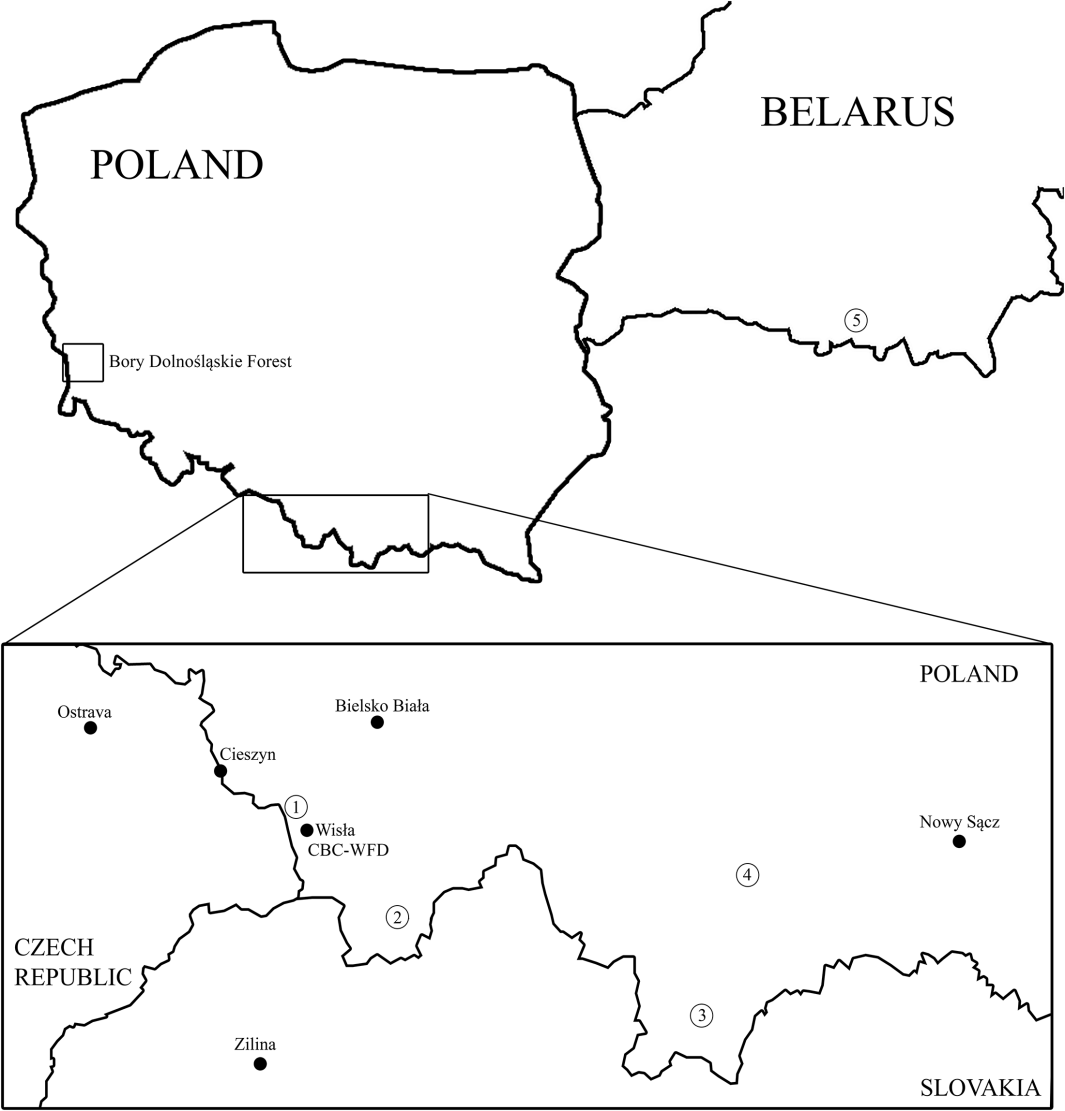
Localization of the Bory Dolnośląskie Forest, Capercaillie Breeding Centre Wisła Forestry District (CBC-WFD) and base population localities for founder individuals (with its number) in CBC-WFD– 1. Czantoria Mountain (1 individual), 2. Ujsoły (2 ind.), 3.Tatry Mountains (1 ind.), 4. Turbacz Mountain (6 ind.), 5. Lielcycy Forestry (7 ind.)

In total, blood stains from 42 individuals from CBC-WFD were collected by a veterinarian from the vena brachialis, previously punctured with a needle. A drop of blood was taken on filter paper and dried immediately in the air. Seventeen individuals were founders—they were wild born or hatched from eggs collected in the wild. Founders source population localities are presented in Fig 1 and described in S1 Table. The remaining 25 individuals were born in the CBC-WFD and were members of a breeding flock (all were of the Western Carpathian lineage) (S1 Table). Furthermore, in 2013, 81 faeces samples were collected from Ruszów Forest District in the BDF. Faeces were collected between January and April 2013 and in December 2013 (three rounds in total) and were dried immediately after collection. Dried faeces were stored in tubes with silica gel until delivery to the laboratory.

### Genetic analysis

#### DNA isolation

DNA from blood stains was extracted with DNeasy DNA Extraction Kit (Qiagen) according to the producer’s manual. DNA from faeces was extracted with the DNA Stool Kit (Qiagen) according to the manufacturer’s instructions modified according to Segelbacher [25] and Regnaut et al. [26].

#### Microsatellites

To determine the genetic variation within nuclear DNA markers, a polymorphism of nine microsatellite loci was analysed among wild and captive populations. Selected loci were previously described by Segelbacher [27] and Piertney and Höglund [28]. All used microsatellites are presented in S2 Table. Amplification was performed in two multiplex reactions, (i) TUT1-TUT4, and (ii) BG10, BG12, BG14-BG16, with a Qiagen Multiplex PCR Kit (in 10 μl volume) according to the producer’s instructions (S2 Table). All PCR reactions had negative controls and were carried out with aerosol-resistant filter pipette tips, and all equipment had been exposed to UV radiation prior to use. All non-invasive samples were genotyped at least twice (beginning with DNA isolation) and 15% of samples isolated from blood stains were re-genotyped to estimate the genotyping errors. After amplification, length of the products was read using an ABI 3730 sequencer and determined using GeneMarker 2.4.2 (available at www.softgenetics.com). Then, the results were developed in the package Microsatellite Toolkit for Excel [29], in which expected and observed heterozygosity across loci were calculated. To estimate the number of alleles per locus, deviations from Hardy-Weinberg equilibrium (HWE) and inbreeding coefficient (F_IS_), Genepop 4.1.4 was used [30]. As a correction for multiple substitutions, False Discovery Rate (FDR) analysis was applied [31]. Furthermore, to estimate null allele frequency (NAF) we used the maximum likelihood approach implemented in ML-NullFreq [32]. Gimlet 1.3.3 software [33] was used to estimate Probability of Identity (P_ID_), to find matching genotypes and to estimate genotyping errors in faeces samples.

As we wanted to analyse the genetic relationship between CBC-WFD and BDF populations, the Bayesian approach implemented in STRUCTURE 2.3.4 [34–36] was applied to obtain the probability of individual membership in a distinct nuclear genetic cluster within those two populations. Correlated allele frequencies with an admixture model were used, with informing the prior about localization of samples (we used a founder lineage (Belarusian or Western Carpathian) as prior information). Analysis were performed with K varying from 1 to 15 and ten replicates for each K, with 200 000 burn-in and 1 000 000 replicates after it. To analyse STRUCTURE results we combined the Delta K method [37] with standard prediction of K based on the plotted mean ln probability of K (L(K)). Both plots (Delta K and L(K)) were calculated using Structure Harvester [38]. We used the online tool Clumpak [39] and implemented in it Clumpp [40] and Distruct [41] to estimate pairwise similarities between runs and to visualize the most probable clustering scheme.

#### Mitochondrial loci

To determine the genetic variability within the mitochondrial DNA and to infer phylogenetic relationships between *T*. *urogallus* from our study and other parts of its Eurasian range, a fragment (757 bp) of the mitochondrial control region (CR) was amplified and sequenced. A modified light primer from Segelbacher et al. [13] was used (CR_F 5’-TTGTTCTCAACTACAGGAAC-3’) along with a newly designed primer (CR_R 5’-TCCGACAAGCATTCACTAAA-3’). PCR conditions were as follows: 95°C– 1 min (95°C– 30 s, 52°C– 45 s, 72°C– 30 s) x 35, 72°C– 5 min. After amplification, DNA was sequenced in both directions using an ABI 3730 sequencer.

Sequences from both primers were aligned using Bioedit [42] and a consensus sequence was created for each individual. Next, sequences from all individuals were aligned with MUSCLE [43], then cleaved to obtain uniform alignment blocks. DnaSP [44] was used to estimate the number of haplotypes, haplotype diversity (H_D_), nucleotide diversity (N_D_) and the number of polymorphic sites.

Phylogenetic analyses of all haplotypes from the present study and homological DNA sequences from other representatives of *T*. *urogallus* along with two rooting sequences (*Lyrurus tetrix* and *Tetrao parvirostris*) (S3 Table) were carried out with a maximum likelihood (ML) and Bayesian approach (BA). BA were performed in MrBayes 3.2 [45] and ML analyses in morePhyML 1.14 [46] based on PhyML 3.0 [47]. The data set was analysed with the model HKY +I—as the best-fit substitution model selected with jModelTest 2.1.4 [48,49]. In MrBayes, two independent runs starting from random trees were applied, each using eight Markov chains. Trees were sampled every 200 generations for 20,000,000 generations (with 25% burn-in) and analyses were finished when the average standard deviation of split frequencies for the set of trees excluding burn-in was stabilized at a level far below 0.01.

## Results and Discussion

### Genotyping

Seventy-two out of 81 (88.9%) faeces samples, after DNA isolation, were of proper quality and sufficient quantity of DNA to reach ≥ 7 microsatellite loci per sample. The probability that two unrelated individuals would have the same genotype (P_ID_) was low (4.64 ×10^−8^), as was the probability that siblings would share the same genotype (8.71 ×10^−4^). Thus, we treated samples with the same genotype as one individual and, after removing identical genotypes, the remaining 62 individuals were used for further analysis. All duplicates had the same mtDNA haplotypes and were collected in the same locality. The mean scoring error rate across PCRs of faeces samples was 0.07 for allele dropout and 0 for false allele. Across all loci, the mean error rate for faeces samples was estimated as 0.08 and for blood samples results in both replications were the same for all individuals, i.e. we found no evidence for genotyping errors in blood samples.

In both wild and captive populations, deviations from HWE were found for several loci—four in the CBC-WFD and seven in the BDF (Table 1). Both of these populations were created from genetically distinct individuals originating from different localities and hence are genetically structured, which results in lack of HWE. We found no signs of a null allele in the reintroduced population, but two loci in the captive population had a high NAF: BG10 and BG12, 0.307 and 0.243 respectively (Table 1). As DNA quality isolated from blood stains was very good, the most common reason for presence of a null allele was unlikely. Other factors, apart from low DNA quality, which result in high NAF may be the Wahlund effect or inbreeding [50]. Nevertheless, if structure presence and inbreeding would have influenced NAF, it should have influenced all loci equally and not only the two. The last factor affecting NAF may be sex linkage of the used loci. To analyse the influence of the sex factor, we divided the breeding flock into males and females and repeated the analysis for them separately. For males the NAF did not exceeded 0.1 in any locus and heterozygosities for both loci were as follows: BG10: H_E_ = 0.583, H_O_ = 0.5, BG12: H_E_ = 0.752, H_O_ = 0.706. For females, observed heterozygosities for BG10 and BG12 were 0 and expected heterozygosities were 0.695 for BG10 and 0.686 for BG12. The above results clearly indicated that both loci (BG10 and BG12) are sex linked and as such should be eliminated from any population analyses of capercaillie. Furthermore, we checked all other loci used in our study for sex linkage but we found no evidence for sex linkage for any other microsatellite locus.

**Table 1:** Microsatellite diversity parameters of captive and reintroduced populations for all *loci*. Allele number (N), expected (H_E_) and observed (H_O_) heterozygosity, inbreeding coefficient (F_IS_), HWE analysis p-value and null allele frequency (NAF).

	CBC-WFD						BDF					
	N	H_E_	H_O_	F_IS_	HWE p-value	NAF	N	H_E_	H_O_	F_IS_	HWE p-value	NAF
BG10	4	0.652	0.195	0.703	**0.000**	0.307	3	0.652	0.820	-0.259	**0.033**	0.000
BG12	5	0.713	0.286	0.602	**0.000**	0.243	7	0.657	0.737	-0.123	**0.002**	0.000
BG14	5	0.720	0.857	-0.193	0.130	0.000	4	0.655	1.000	-0.533	**0.000**	0.000
BG15	5	0.753	0.810	-0.076	0.081	0.000	5	0.678	0.968	-0.431	**0.000**	0.000
BG16	8	0.795	0.738	0.073	**0.003**	0.038	7	0.681	0.871	-0.281	**0.000**	0.000
TUT1	7	0.727	0.571	0.216	**0.003**	0.099	4	0.655	0.742	-0.134	0.106	0.000
TUT2	6	0.697	0.786	-0.130	0.947	0.000	5	0.553	0.603	-0.092	0.137	0.000
TUT3	7	0.799	0.786	0.017	0.238	0.000	6	0.748	0.911	-0.220	**0.000**	0.000
TUT4	6	0.796	0.854	-0.074	0.783	0.000	6	0.754	0.949	-0.262	**0.000**	0.000
Average	5.9	0.739	0.654	0.117			5.2	0.670	0.844	-0.262		
Average*	6.3	0.755	0.772	-0.022			5.3	0.675	0.863	-0.285		

* Without BG10/BG12

Bold—significant after FDR correction.

Realistic results in non-invasive samples are difficult to obtain as genotyping errors always more or less affect their quality. To avoid erroneous interpretation of results, one has to adapt laboratory procedures and quality control to low DNA samples [51]. Even though researchers follow procedures for non-invasive samples, in some cases the results are still flawed. BG10 and BG12 loci have already been used in population genetic analysis [52,53], and in both papers the authors did not exclude them from the dataset. As all present western and central European capercaillie populations have experienced significant population decline, in many cases analysed loci deviate from HWE, showing the presence of null alleles, and it is very difficult to determine the cause of this situation. Thus, in our opinion all analysed loci should always be checked for sex linkage presence (if there is such a possibility—there is sex information) as it disrupts allele frequency, which is the basis for further population analysis.

### Genetic diversity

Genetic diversity parameters, revealed with microsatellites in populations from the CBC-WFD and the BDF, are presented in Table 1. Average allele number and expected heterozygosity were higher in the captive population (N_A_: 6.3 vs. 5.3; H_E_: 0.755 vs. 0.675). On the other hand, average observed heterozygosity was higher in the wild population (0.772 vs. 0.863). Both populations (with both sex-linked loci excluded) were characterized by excess of heterozygotes. What we found interesting, when we analysed both populations without eliminating BG10/BG12 loci, the wild population presented only slightly different diversity parameters, while in the breeding one observed heterozygosity and F_IS_ differed widely (especially F_IS:_ 0.117 vs. -0.022). This is probably the result of different sex ratios in the two populations. In the CBC-WFD the male-to-female ratio is 1:2.2. In the reintroduced population the sex ratio is unknown, but in other wild capercaillie populations there is usually a majority of males: 1:1.6 [53] and 1:1.5 [52]. That explains why in wild populations it is difficult to detect sex linkage without separate analysis of males and females, as male birds are homogametic (ZZ).

When compared to other wild capercaillie populations, the population newly reintroduced in the BDF was characterised by high values of diversity parameters revealed with nuclear DNA. An isolated, Cantabrian capercaillie population presented low genetic diversity (H_E_ = 0.5, N_A_ = 3.4 [52]; H_E_ = 0.57, N_A_ = 11.75 [14]). Selected European populations described by Segelbacher et al. [7] had diversity parameters in the range 2.8–6.3 (N_A_) and 0.45–0.66 (H_E_). Finally, a Polish *T*. *urogallus* population described by Rutkowski et al. [9] revealed average H_E_ equal to 0.585 and average allele number equal to 4.45, while the former BDF population had H_E_ = 0.59 and N_A_ = 5. Thus, diversity parameters presented by us (N_A_ = 5.3 and H_E_ = 0.675) in the newly created BDF population indicate high genetic variation, which may result in adaptive potential necessary for population restoration and survival.

**Fig 2:**
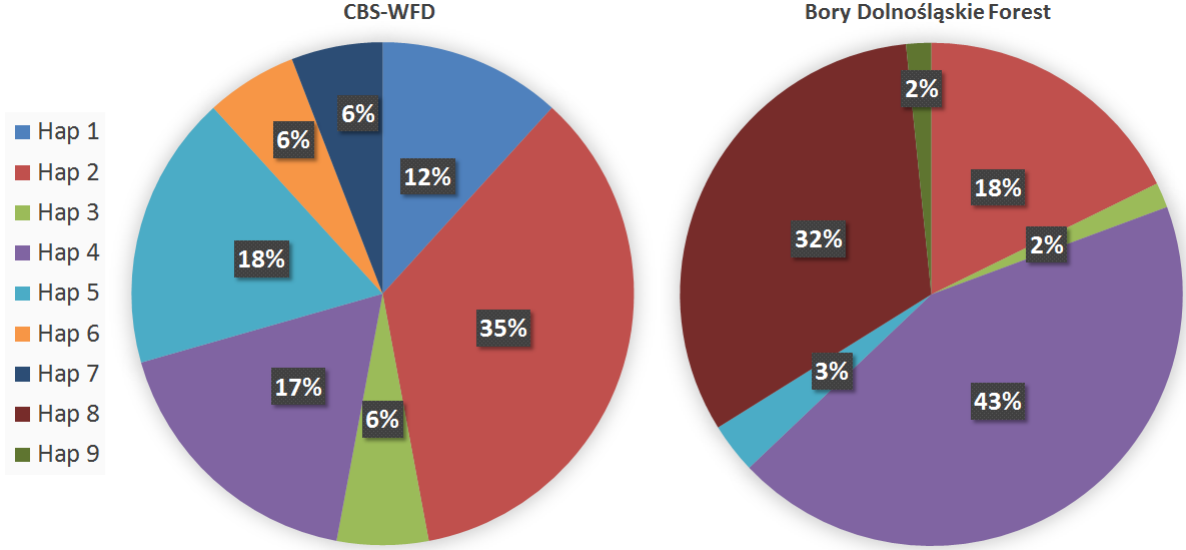
Frequency of the mtDNA haplotypes among CBC-WFD founders’ population and BDF reintroduced population.

Control region amplification revealed the presence of six mtDNA haplotypes in the BDF and seven among the captive founder population (Fig 2) (we did not sequence offspring in the captive population as they share the same mtDNA with their mothers). In total, nine different mtDNA haplotypes were present (GenBank accession numbers: KT223509-KT223517) and among them four were common for both populations, three were unique for the captive population and two for the wild population (S1 Table). Despite the much lower number of analysed individuals (17 captive vs. 62 reintroduced), all mtDNA diversity parameters were higher in the founder captive population (Table 2).

**Table 2:** mtDNA variability parameters in captive and reintroduced populations. Number of sequences (N_S_), sequence length (L), number of polymorphic sites (N_P_), number of haplotypes (N_H_), haplotype diversity (H_D_) and nucleotide diversity (N_D_).

Population	N_S_	L	N_P_	N_H_	H_D_	N_D_
Bory Dolnośląskie Forest	62	757	7	6	0.684 (±0.034)	0.003 (±0.0009)
CBC-WFD	17	757	9	7	0.838 (±0.063)	0.0043 (±0.001)

Reintroduced population mtDNA diversity indices obtained in our study (H_D_ = 0.684, N_D_ = 0.003) had lower values when compared to average nucleotide and haplotype diversity indices in three groups of capercaillie analysed by Duriez et al. [10]. But when we compared mtDNA diversity indices of BDF population with seven individuals from the same region (Czech Republic—Poland) we found that haplotype diversity is higher in reintroduced population (0.524 vs 0.684) [10]. This suggests that BDF population is in better “genetic condition” than other local populations but also indicates that the haplotype diversity present in BDF is still on the same, low level, as in endemic population from Pyrenees [10].

### Genetic relationship between capercaillie from CBC-WFD and BDF

**Fig 3:**
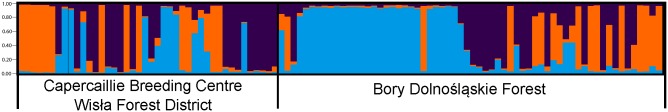
Clustering results of combined CBC-WFD and BDF population revealed with STRUCTURE. Three distinct genetic clusters were present: one consisted of individuals from Belarussian lineage (orange) and two of Western Carpathian lineage (blue and violet). Each line represents one individual and the amount of the colour indicates estimated membership of the individual in a distinct genetic cluster.

STRUCTURE analysis showed both the highest value of mean estimated Ln P(K) and the lowest standard deviation for K = 3. The delta K method confirmed those results and also indicated K = 3 as the most probable number of distinct genetic clusters among all analysed individuals. In the CBC-WFD founder population, three clusters consisted of: i) cluster one—all individuals from the Belarusian lineage (except female 25, which was a hybrid between clusters two and three) and female 48, which was of Western Carpathian origin but was clustered with individuals from Belarus; ii) clusters two and three—individuals from the Western Carpathian lineage. In both populations (wild and captive) we found the presence of all three genetic clusters (Fig 3). In the whole CBC-WFD population 31% of individuals belonged to cluster one, 21% to cluster two and 36% to cluster three. The remaining 12% of individuals within CBC-WFD capercaillie were hybrids. In the BDF population, the most common genetic cluster was cluster two (40%), then cluster three (23%) and cluster one (21%). Sixteen percent of individuals among BDF individuals were hybrids.

The most frequent mtDNA haplotypes in the reintroduced population were haplotypes no. four (43%), eight (32%) and two (18%), while in the captive founder population they were haplotypes no. two (35%), four (17%) and one (12%) (Fig 2). In the CBC-WFD founder population, haplotype no. one was found only among individuals from Belarus, haplotype no. two was most common among grouse from Belarus, being found only in two individuals outside this group (female no. 25 and male no. 20), and the rest of haplotypes were present only among individuals from the Western Carpathian lineage.

Both analysed populations (reintroduced and captive) presented the same genetic structure revealed with microsatellites. Three distinct genetic clusters differed however with frequencies in both populations. Thus, based on microsatellites we may state that both analysed groups of grouse were genetically similar. When we compared both populations with mtDNA, we found much greater disparities. Thirty-four percent of individuals from the BDF had two mtDNA haplotypes (no. eight and nine) not present in the captive population, which suggests that at least 34% of wild individuals were reared or are offspring of females reared outside the CBC-WFD.

### Phylogenetic analysis

Both ML and BA phylogenetic trees revealed the same topology showing two distinct clades, Eurasian and Cantabrian (S1 Fig), as found in other studies [10–12,54]. All haplotypes from the present study were located in the Eurasian clade. Unfortunately, due to the lack of resolution resulting from the short DNA sequence used in phylogenetic analysis (CR sequences obtained from GenBank were shorter than DNA in this study, and after alignment the set of sequences had only 369 bp), we were not able to clarify relationships between particular haplotypes within the Eurasian clade. Bayesian posterior probabilities and SH-like branch support for nodes within clades were low and thus not significant. Nevertheless, all analysed individuals (both from Belarus and Carpathian) belonged to the same mitochondrial lineage, which suggests that they represent the same evolutionarily significant units (ESU) and as such may be used together in the reintroduction process.

### Pedigree-based breeding reliability

When we compared pedigree data with genetic based information, we found several inaccuracies. First, some founder individuals, which should (according to breeding documentation) derive from a certain genetic line (Belarusian or Western Carpathian), did not derive from those lines. Second, captive population 1^st^ generation offspring derived from the Western Carpathian lineage turned out to have in part or in whole genotypes specific to capercaillie from Belarus. This may be the result of wrong assignment of founder individuals into genetic lineages or an inexact process of egg incubation and chick rearing. As all captive individuals belong to the same phylogenetic lineage, Eurasian, in this case crossing of individuals with different origin did not have negative consequences. But, once more it shows how important it is to implement genetic tools in captive breeding. Genetic based selection provides solid information which will ensure proper selection of animals for breeding and thereby ensure the preservation of a high level of biodiversity in the breeding flock.

In conclusion, the genetic characteristics of both reintroduced BDF and captive CBC-WFD populations of Western capercaillie showed a high level of diversity. The main objective of captive breeding is to create a self-sustainable population capable of producing progeny that can be further used for reintroduction [5]. Thus, we showed that by this high level of biodiversity the CBC-WFD captive population poses genetic potential to be such a self-sustainable population and valuable source of individuals for reintroduction. The high level of diversity among individuals in the BDF population is a good predictor for the future, as it shows the first positive results of Bory Dolnośląskie Forest actions and leads to the conclusion that their continuation will help to fully rebuild the capercaillie population in this forest district. Thus, our results will be the basis to facilitate monitoring of the reintroduction process in the future.

## Supporting Information

S1 Table List of individuals/samples used in the present study, along with sex information, samples localization and mtDNA haplotype/microsatellite cluster belonging. (PDF)

S2 Table Microsatellite set used in the presented analysis. Names, primers and fluorescent dyes with annealing temperatures are presented.
(PDF)

S3 Table Sequences possessed from Genbank and used in phylogenetic analysis along with accession number and author. (PDF)

S1 Fig Bayesian phylogenetic tree of CR haplotypes from this analysis connected with haplotypes sequences from Genbank (S3 Table). Numbers along nodes are their Bayesian posterior probability values (BA, above) and aLRT values (ML, below).
(TIFF)
