# Supplementary material for: Reintroduction of the European Capercaillie from the Capercaillie Breeding Centre in Wisła Forest District: Genetic Assessments of Captive and Reintroduced Populations
Source: PLoS One. 2015 Dec 18;10(12):e0145433. doi: 10.1371/journal.pone.0145433 (PMC4684292; doi:10.1371/journal.pone.0145433)
Supplement: S1 Table — (PDF) [file pone.0145433.s001.pdf]

Group	Pedigree/ Lab no.	Sex	Location	mtDNA haplotype	Microsatellite cluster
CBC-WFD founders	6	F	Belarus, Lielcycy Forestry	Hap 1	1
	9	M	Belarus, Lielcycy Forestry	Hap 2	1
	16	M	Belarus, Lielcycy Forestry	Hap 1	1
	19	M	Poland. Ujsoły	Hap 3	2
	20	M	Poland, Czantoria Mountain	Hap 2	2
	21	F	Belarus, Lielcycy Forestry	Hap 2	1
	22	F	Belarus, Lielcycy Forestry	Hap 2	1
	23	F	Belarus, Lielcycy Forestry	Hap 2	1
	24	F	Poland, Turbacz Mountain	Hap 4	2
	25	F	Belarus, Lielcycy Forestry	Hap 2	hybrid
	25	M	Poland. Ujsoły	Hap 5	3
	46	F	Poland, Turbacz Mountain	Hap 6	3
	47	F	Poland, Turbacz Mountain	Hap 5	3
	48	F	Poland, Turbacz Mountain	Hap 4	1
	49	M	Poland, Turbacz Mountain	Hap 7	3
	50	M	Poland, Turbacz Mountain	Hap 4	3
	51	M	Poland, Tatry Mountains	Hap 5	3
CBC-WFD offspring	26S	F	CBC-WFD offspring	-	1
	27S	F	CBC-WFD offspring	-	3
	28S	F	CBC-WFD offspring	-	1
	29S	F	CBC-WFD offspring	-	2
	30S	F	CBC-WFD offspring	-	hybrid
	52S	F	CBC-WFD offspring	-	hybrid
	53S	F	CBC-WFD offspring	-	2
	54S	F	CBC-WFD offspring	-	2
	55S	F	CBC-WFD offspring	-	2
	56S	F	CBC-WFD offspring	-	1
	58S	M	CBC-WFD offspring	-	1
	66S	F	CBC-WFD offspring	-	hybrid
	67S	F	CBC-WFD offspring	-	2
	68S	F	CBC-WFD offspring	-	hybrid
	70S	F	CBC-WFD offspring	-	3
	71S	F	CBC-WFD offspring	-	3
	73S	M	CBC-WFD offspring	-	1
	74S	M	CBC-WFD offspring	-	1
	79S	M	CBC-WFD offspring	-	3
	80S	F	CBC-WFD offspring	-	3
	81S	F	CBC-WFD offspring	-	3
	82S	F	CBC-WFD offspring	-	3
	83S	F	CBC-WFD offspring	-	2
	84S	F	CBC-WFD offspring	-	3
	85S	M	CBC-WFD offspring	-	3
	1F	-	Bory Dolnośląskie Forest	Hap 8	hybrid
	2F	-	Bory Dolnośląskie Forest	Hap 8	1

Non invasive samples from BDF

3F	-	Bory Dolnośląskie Forest	Hap 8	3
4F	-	Bory Dolnośląskie Forest	Hap 4	2
5F	-	Bory Dolnośląskie Forest	Hap 8	2
6F	-	Bory Dolnośląskie Forest	Hap 2	2
7F	-	Bory Dolnośląskie Forest	Hap 3	2
8F	-	Bory Dolnośląskie Forest	Hap 4	2
9F	-	Bory Dolnośląskie Forest	Hap 9	2
10F	-	Bory Dolnośląskie Forest	Hap 4	2
11F	-	Bory Dolnośląskie Forest	Hap 2	2
12F	-	Bory Dolnośląskie Forest	Hap 8	2
13F	-	Bory Dolnośląskie Forest	Hap 8	2
14F	-	Bory Dolnośląskie Forest	Hap 4	2
15F	-	Bory Dolnośląskie Forest	Hap 2	2
16F	-	Bory Dolnośląskie Forest	Hap 8	2
17F	-	Bory Dolnośląskie Forest	Hap 2	2
18F	-	Bory Dolnośląskie Forest	Hap 4	2
19F	-	Bory Dolnośląskie Forest	Hap 4	2
20F	-	Bory Dolnośląskie Forest	Hap 4	2
21F	-	Bory Dolnośląskie Forest	Hap 4	2
22F	-	Bory Dolnośląskie Forest	Hap 4	2
23F	-	Bory Dolnośląskie Forest	Hap 8	2
24F	-	Bory Dolnośląskie Forest	Hap 4	1
25F	-	Bory Dolnośląskie Forest	Hap 8	2
26F	-	Bory Dolnośląskie Forest	Hap 4	2
27F	-	Bory Dolnośląskie Forest	Hap 8	2
28F	-	Bory Dolnośląskie Forest	Hap 8	2
29F	-	Bory Dolnośląskie Forest	Hap 4	2
30F	-	Bory Dolnośląskie Forest	Hap 4	hybrid
31F	-	Bory Dolnośląskie Forest	Hap 2	hybrid
32F	-	Bory Dolnośląskie Forest	Hap 4	3
33F	-	Bory Dolnośląskie Forest	Hap 8	3
34F	-	Bory Dolnośląskie Forest	Hap 4	3
35F	-	Bory Dolnośląskie Forest	Hap 8	3
36F	-	Bory Dolnośląskie Forest	Hap 4	3
37F	-	Bory Dolnośląskie Forest	Hap 2	3
38F	-	Bory Dolnośląskie Forest	Hap 4	hybrid
39F	-	Bory Dolnośląskie Forest	Hap 4	3
40F	-	Bory Dolnośląskie Forest	Hap 8	3
41F	-	Bory Dolnośląskie Forest	Hap 4	1
42F	-	Bory Dolnośląskie Forest	Hap 4	1
43F	-	Bory Dolnośląskie Forest	Hap 4	3
44F	-	Bory Dolnośląskie Forest	Hap 8	hybrid
45F	-	Bory Dolnośląskie Forest	Hap 8	hybrid
46F	-	Bory Dolnośląskie Forest	Hap 2	hybrid
47F	-	Bory Dolnośląskie Forest	Hap 8	hybrid
48F	-	Bory Dolnośląskie Forest	Hap 8	1
49F	-	Bory Dolnośląskie Forest	Hap 2	3
50F	-	Bory Dolnośląskie Forest	Hap 4	1

51F	-	Bory Dolnośląskie Forest	Hap 4	1
52F	-	Bory Dolnośląskie Forest	Hap 4	3
53F	-	Bory Dolnośląskie Forest	Hap 5	hybrid
54F	-	Bory Dolnośląskie Forest	Hap 2	3
55F	-	Bory Dolnośląskie Forest	Hap 8	1
56F	-	Bory Dolnośląskie Forest	Hap 2	3
57F	-	Bory Dolnośląskie Forest	Hap 5	hybrid
58F	-	Bory Dolnośląskie Forest	Hap 8	1
59F	-	Bory Dolnośląskie Forest	Hap 4	1
60F	-	Bory Dolnośląskie Forest	Hap 2	1
61F	-	Bory Dolnośląskie Forest	Hap 4	1
62F	-	Bory Dolnośląskie Forest	Hap 4	1
