# Supplementary material for: Reintroduction of the European Capercaillie from the Capercaillie Breeding Centre in Wisła Forest District: Genetic Assessments of Captive and Reintroduced Populations
Source: PLoS One. 2015 Dec 18;10(12):e0145433. doi: 10.1371/journal.pone.0145433 (PMC4684292; doi:10.1371/journal.pone.0145433)
Supplement: S2 Table — Names, primers and fluorescent dyes with annealing temperatures are presented. (PDF) [file pone.0145433.s002.pdf]

No	*Locus*	Primers	Primers sequence	Dye	Annealing temperature
1	TUT 1	TUT1_F	GGTCTACATTTGGCTCTGACC	FAM	60º C
		TUT1_R	ATATGGCATCCCAGCTATGG		
2	TUT 2	TUT2_F	CCGTGTCAAGTTCTCCAAAC	FAM	
		TUT2_R	TTCAAAGCTGTGTTTCATTAGTTG		
3	TUT 3	TUT3_F	CAGGAGGCCTCAACTAATCACC	CY3	
		TUT3_R	CGATGCTGGACAGAAGTGAC		
4	TUT 4	TUT4_F	GAGCATCTCCCAGAGTCAGC	HEX	
		TUT4_R	TGTGAACCAGCAATCTGAGC		
5	BG 10	BG10_F	ATGTTTCATGTCTTCTGGAATAG	HEX	58º C
		BG10_R	ATTTGGTTAGTAACGCATAAG		
6	BG 12	BG12_F	TCTCCTTCTAAACCAGTCATTC	CY3	
		BG12_R	TAGTTTCCACAGAGCACATTG		
7	BG 14	BG14_F	ATCCTACTGAACAAAATATCTGC	FAM	
		BG14_R	TATGCAGGTAGGTAGTGAGAGAG		
8	BG 15	BG15_F	AAATATGTTTGCTAGGGCTTAC	FAM	
		BG15_R	TACATTTTTCATTGTGGACTTC		
9	BG 16	BG16_F	GTCATTAGTGCTGTCTGTCTATCT	HEX	
		BG16_R	TGCTAGGTAGGGTAAAAATGG		
