# Supplementary material for: Reintroduction of the European Capercaillie from the Capercaillie Breeding Centre in Wisła Forest District: Genetic Assessments of Captive and Reintroduced Populations
Source: PLoS One. 2015 Dec 18;10(12):e0145433. doi: 10.1371/journal.pone.0145433 (PMC4684292; doi:10.1371/journal.pone.0145433)
Supplement: S3 Table — (PDF) [file pone.0145433.s003.pdf]

No.	Species	Accession number	Authors
1	Tetrao urogallus	HQ852192	Bajc,M.
2	Tetrao urogallus	AY750940	De la Cruz-Cardiel,P.J., Alda,F. and Doadrio,I.
3	Tetrao urogallus	AY750955	De la Cruz-Cardiel,P.J., Alda,F. and Doadrio,I.
4	Tetrao urogallus	AY750938	De la Cruz-Cardiel,P.J., Alda,F. and Doadrio,I.
5	Tetrao urogallus	AY750951	De la Cruz-Cardiel,P.J., Alda,F. and Doadrio,I.
6	Tetrao urogallus	AY750946	De la Cruz-Cardiel,P.J., Alda,F. and Doadrio,I.
7	Tetrao urogallus	AY750941	De la Cruz-Cardiel,P.J., Alda,F. and Doadrio,I.
8	Tetrao urogallus	AY750942	De la Cruz-Cardiel,P.J., Alda,F. and Doadrio,I.
9	Tetrao urogallus	AY750954	De la Cruz-Cardiel,P.J., Alda,F. and Doadrio,I.
10	Tetrao urogallus	AY580996	Liukkonen-Anttila,T., Ratti,O., Kvist,L., Helle,P. and Orell,M.
11	Tetrao urogallus	AY581009	Liukkonen-Anttila,T., Ratti,O., Kvist,L., Helle,P. and Orell,M.
12	Tetrao urogallus	AY581012	Liukkonen-Anttila,T., Ratti,O., Kvist,L., Helle,P. and Orell,M.
13	Tetrao urogallus	HQ852178	Bajc,M.
14	Tetrao urogallus	AY581014	Liukkonen-Anttila,T., Ratti,O., Kvist,L., Helle,P. and Orell,M.
15	Tetrao urogallus	AY581029	Liukkonen-Anttila,T., Ratti,O., Kvist,L., Helle,P. and Orell,M.
16	Tetrao urogallus	AY581033	Liukkonen-Anttila,T., Ratti,O., Kvist,L., Helle,P. and Orell,M.
17	Tetrao urogallus	AY581034	Liukkonen-Anttila,T., Ratti,O., Kvist,L., Helle,P. and Orell,M.
18	Tetrao urogallus	AY581035	Liukkonen-Anttila,T., Ratti,O., Kvist,L., Helle,P. and Orell,M.
19	Tetrao urogallus	AY581036	Liukkonen-Anttila,T., Ratti,O., Kvist,L., Helle,P. and Orell,M.
20	Tetrao urogallus	DQ398961	Rodriguez Munoz,R., Mirol,P.M., Segelbacher,G., Fernandez,A. and Tregenza,T.
21	Tetrao urogallus	HQ852176	Bajc,M.
22	Tetrao urogallus	HQ852175	Bajc,M.
23	Tetrao urogallus	HQ852179	Bajc,M.
24	Tetrao urogallus	HQ852180	Bajc,M.
25	Tetrao urogallus	HQ852177	Bajc,M.
26	Tetrao urogallus	DQ398967	Rodriguez Munoz,R., Mirol,P.M., Segelbacher,G., Fernandez,A. and Tregenza,T.
27	Tetrao urogallus	DQ398971	Rodriguez Munoz,R., Mirol,P.M., Segelbacher,G., Fernandez,A. and Tregenza,T.
28	Tetrao urogallus	DQ307395	Duriez,O., Sachet,J.-M., Menoni,E., Pidancier,N., Miquel,C. and Taberlet,P.
29	Tetrao urogallus	DQ307396	Duriez,O., Sachet,J.-M., Menoni,E., Pidancier,N., Miquel,C. and Taberlet,P.
30	Tetrao urogallus	DQ307397	Duriez,O., Sachet,J.-M., Menoni,E., Pidancier,N., Miquel,C. and Taberlet,P.
31	Tetrao urogallus	DQ307398	Duriez,O., Sachet,J.-M., Menoni,E., Pidancier,N., Miquel,C. and Taberlet,P.
32	Tetrao urogallus	DQ307399	Duriez,O., Sachet,J.-M., Menoni,E., Pidancier,N., Miquel,C. and Taberlet,P.
33	Tetrao urogallus	DQ307407	Duriez,O., Sachet,J.-M., Menoni,E., Pidancier,N., Miquel,C. and Taberlet,P.
34	Tetrao urogallus	DQ307408	Duriez,O., Sachet,J.-M., Menoni,E., Pidancier,N., Miquel,C. and Taberlet,P.
35	Tetrao urogallus	DQ307410	Duriez,O., Sachet,J.-M., Menoni,E., Pidancier,N., Miquel,C. and Taberlet,P.
36	Tetrao urogallus	DQ307411	Duriez,O., Sachet,J.-M., Menoni,E., Pidancier,N., Miquel,C. and Taberlet,P.
37	Tetrao urogallus	DQ307414	Duriez,O., Sachet,J.-M., Menoni,E., Pidancier,N., Miquel,C. and Taberlet,P.
38	Tetrao urogallus	DQ307415	Duriez,O., Sachet,J.-M., Menoni,E., Pidancier,N., Miquel,C. and Taberlet,P.
39	Tetrao urogallus	DQ307416	Duriez,O., Sachet,J.-M., Menoni,E., Pidancier,N., Miquel,C. and Taberlet,P.

40	Tetrao urogallus	DQ307417	Duriez,O., Sachet,J.-M., Menoni,E., Pidancier,N., Miquel,C. and Taberlet,P.
41	Lyrurus tetrix	JX965032	Xu,X.-L., Cai,X.-Q. and Bai,S.-Y.
42	Tetrao parvirostris	AF532462	Drovetski,S.V.
